# Supplementary material for: A method to estimate the contribution of rare coding variants to complex trait heritability
Source: Nat Commun. 2024 Feb 9;15:1245. doi: 10.1038/s41467-024-45407-8 (PMC10858280; doi:10.1038/s41467-024-45407-8)
Supplement: Supplementary file 1 — Supplementary Information [file 41467_2024_45407_MOESM1_ESM.pdf]

Supplementary Information

A Method to Estimate the Contribution of Rare Coding Variants to Complex Trait Heritability

Nazia Pathan,[1,2] Wei Q. Deng,[3,4] Matteo Di Scipio,[1,5] Mohammad Khan,[1,5] Shihong Mao,[1] Robert W. Morton,[1,2] Ricky Lali,[1,6] Marie Pigeyre,[1,5] Michael R. Chong,[1,2,7] Guillaume Paré[1,2,6,7*]

*Corresponding author

[1]Population Health Research Institute, David Braley Cardiac, Vascular and Stroke Research Institute, Hamilton Health Sciences and McMaster University, Hamilton, Canada.
[2]Department of Pathology and Molecular Medicine, McMaster University, Michael G. DeGroote School of Medicine, Hamilton, Canada.
[3]Peter Boris Centre for Addictions Research, St. Joseph's Healthcare Hamilton, Hamilton, Canada.
[4]Department of Psychiatry and Behavioural Neurosciences, McMaster University, Hamilton, Canada.
[5]Department of Medicine, Faculty of Health Sciences, McMaster University, Hamilton, Canada.
[6]Department of Health Research Methods, Evidence, and Impact, McMaster University, Hamilton, Canada.
[7]Thrombosis and Atherosclerosis Research Institute, David Braley Cardiac, Vascular and Stroke Research Institute, Hamilton, Canada.

Supplemental Figures: 11

Supplemental Tables: 7

Corresponding Author:

Guillaume Paré MD, MSc, FRCPC

McMaster University Population Health Research Institute

David Braley Cardiac, Vascular, and Stroke Research Institute

237 Barton Street East – C4 126

E-mail: pareg@mcmaster.ca

Contents

Supplementary Fig.1: Summary of the Rare variant heritability (RARity) estimator pipeline…………………………………………………………… 2

Supplementary Fig.2: Statistical power of RARity……………………… 3

Supplementary Fig.3: Simulation of rare variant heritability estimates under varying LD pruning, fraction of causal genes and variants, and MAF conditions…………………………………………………………………… 4

Supplementary Fig.4: Comparison of RV exome-wide heritability estimates between the sexes for 31 continuous traits. ……………………… 5

Supplementary Fig.5: Comparison of methods to estimate common variant (MAF>0.01) heritability estimates. ………………………………… 6

Supplementary Fig.6: Correlation between the contribution of RV and CV to complex traits heritability. ……………………………………………… 7

Supplementary Fig.7: Impact of pathogenicity scores on variance explained by RVs for 31 complex traits..…………………………………… 8

Supplementary Fig.8: Spline plots for the associations of $\log_{10}$ (Gene length (bp)) with $h^2_{\text{gene-RV}}$. ………………………………………………… 11

Supplementary Fig.9: Spline plots for the associations of evolutionary constraint (LOEUF) with $h^2_{\text{gene-RV}}$. ………………………………………… 12

Supplementary Fig.10: Comparison of RV heritability estimates between the genes encoded in the positive vs negative strands. …………………… 13

Supplementary Fig.11: Impact of block size on RV heritability estimates... 14

Supplementary Table 1: Abbreviations used to describe the phenotypes.… 15

Supplementary Table 2: Medications used for adjusting biomarkers values………………………………………………………………...... 16

Supplementary Table 3: Comparison of RV heritability estimates derived using gene-burden, gene-wise and exome-wide blocks…………………….. 17

Supplementary Table 4: RV heritability by MAF bins calculated using gene-wise blocks……………………………………………………………….. 18

Supplementary Table 5: Rare coding variant heritability as a function of $\log_{10}$(Gene-length)……………………………………………………………... 19

Supplementary Table 6: Rare coding variant heritability as a function of evolutionary constraint..…………………………………………………….. 20

Supplementary Table 7: Heritability of height originating from RVs in selected gene clusters...……………………………………………………... 20

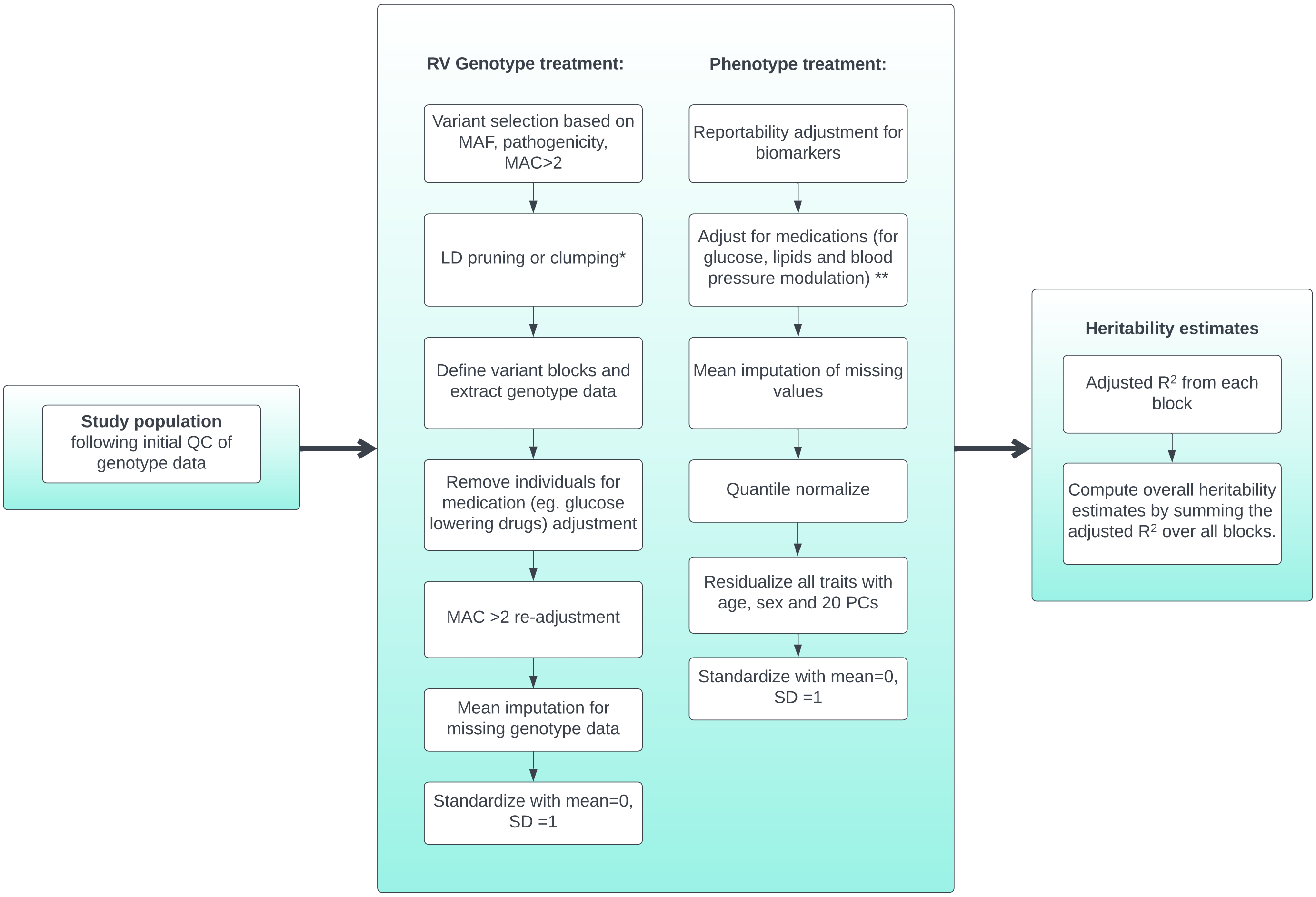

Supplementary Fig.1: Summary of the Rare variant heritability (RARity) estimator pipeline. The RARity pipeline constitutes pre-treatment of the genotype and the phenotype data, followed by application of the statistical model to each block, and finally estimation of the total heritability from all blocks. *The model may be modified to prioritize variants by implementing LD clumping instead of pruning. In addition, the pruning parameters are dependent on the selection of common *vs* rare variants for the analysis. **Adjustment of medications may involve implementing correction factors or removal of individuals using the medications. MAF = minor allele frequency, MAC = minor allele count, LD = linkage disequilibrium, SD = standard deviation, PCs = principal components.

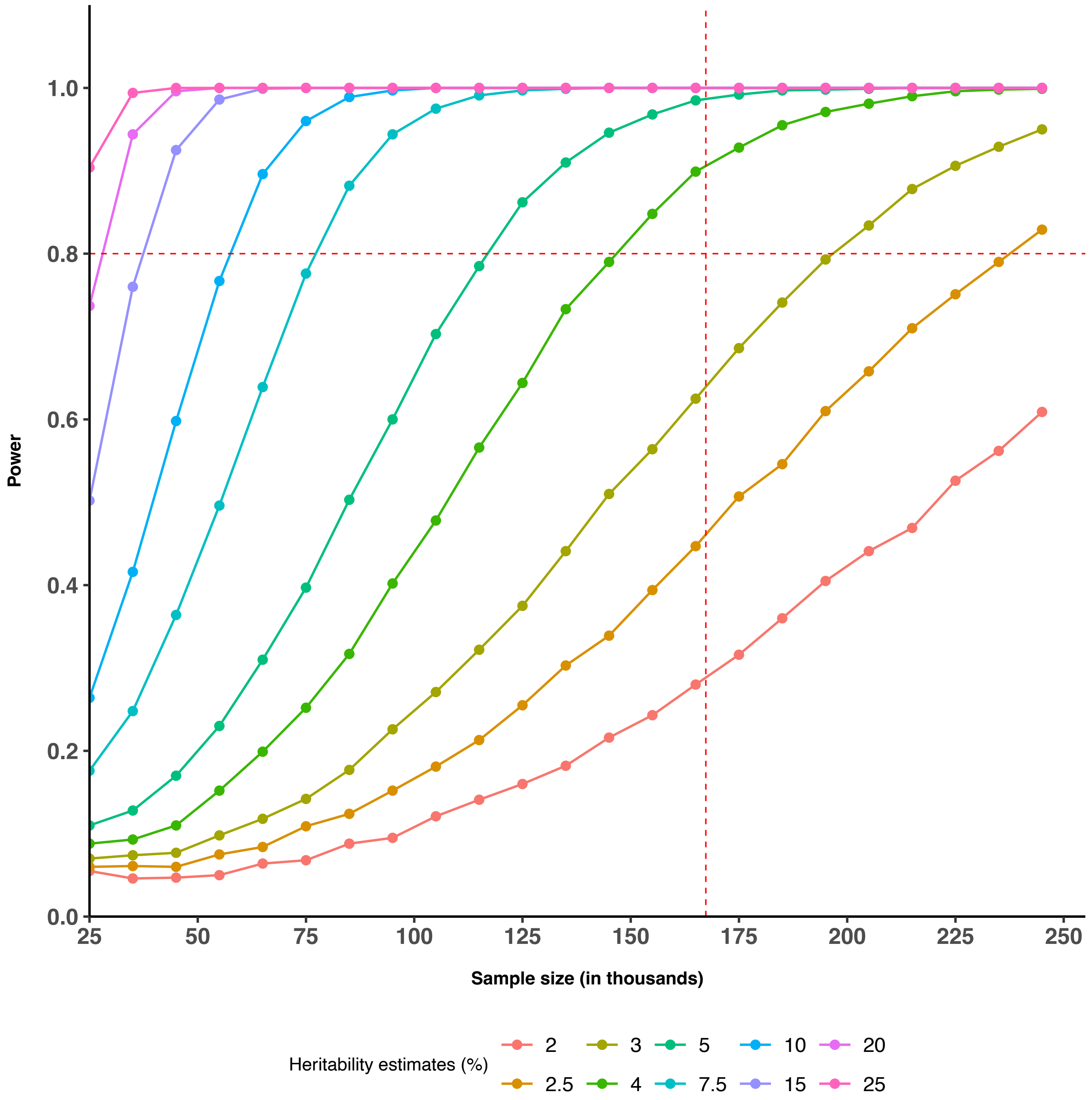

Supplementary Fig.2: Statistical power of RARity. The statistical power of rare coding variant heritability with RARity is displayed as a function of sample size for different levels of RV heritability estimates. Statistical power was estimated empirically from the variance of 10,000 simulated h^2_{RV}, under 230 conditions of sample sizes and true set h^2_{RV}, with each condition being represented by a dot. The red, horizontal dashed line corresponds to an empirical 80% power with alpha-level of 0.05. The red, vertical dashed line marks the sample size used in the current study.

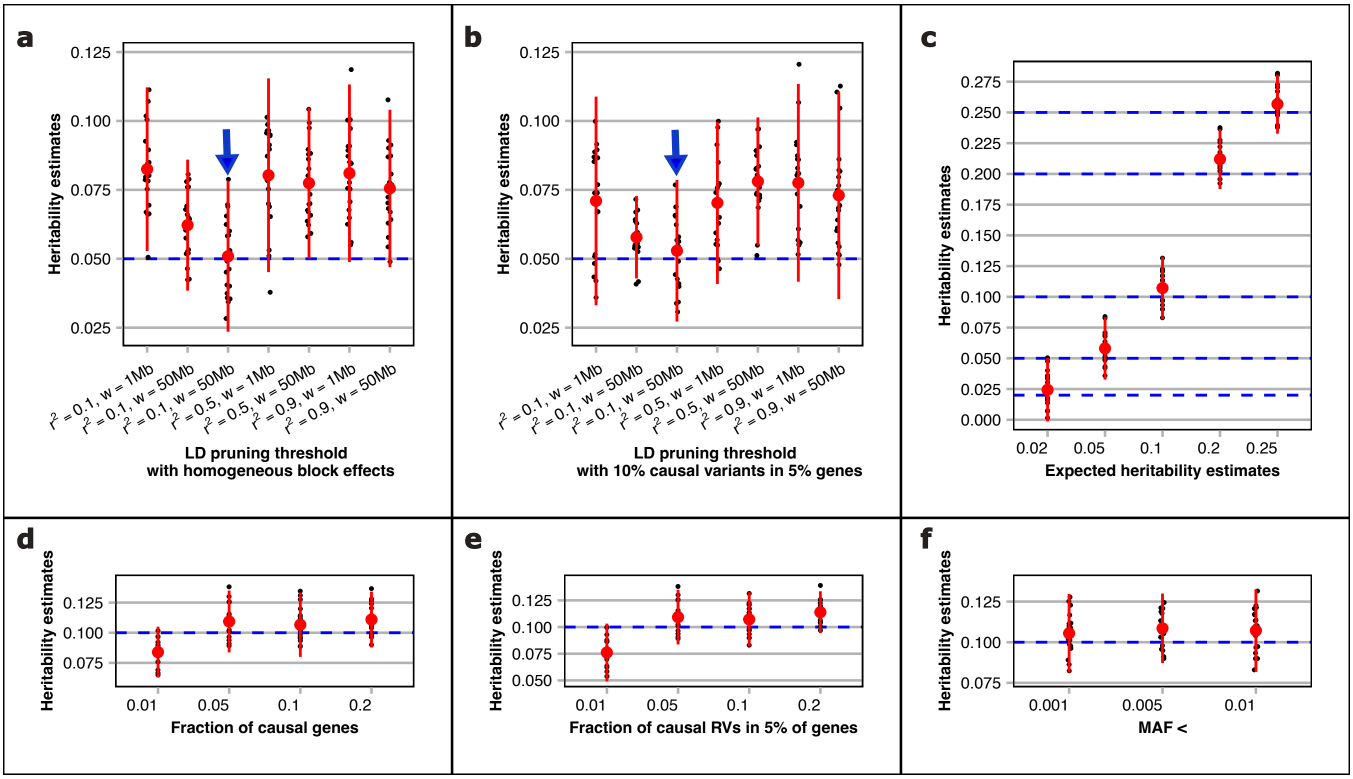

Supplementary Fig.3: Simulation of rare variant heritability estimates under varying LD pruning, fraction of causal genes and variants, and MAF conditions. Calibration of RARity for linkage disequilibrium, where r^2 = 'coefficient of correlation' and w = 'window size' thresholds used for LD pruning, (a) assuming homogenous distribution of heritability across all exome-wide blocks, and (b) assuming 10% of causal variants in 5% genes. In both cases, unbiased estimation of h^2_{RV} was observed when RVs were pruned with $r^2 > 0.1$ within a window size of 50Mb (blue arrows) and is the default LD pruning threshold used for all analyses. Figures (c-f) examined the sensitivity of RARity to varying (c) true h^2_{RV} values; (d) fraction of causal genes with 10% RVs with effects; (e) fraction of causal RVs in 5% of the genes and (f) MAF thresholds assuming 10% causal RVs within 5% of genes has an effect. Each dot in the figure represents a single simulation, with 20 exome-wide simulations performed for each scenario. The red vertical lines represent the 95% CIs, and the blue dashed, horizontal lines represent the true h^2_{RV}. Except for (f), RVs with MAF < 0.01 were selected for all analyses.

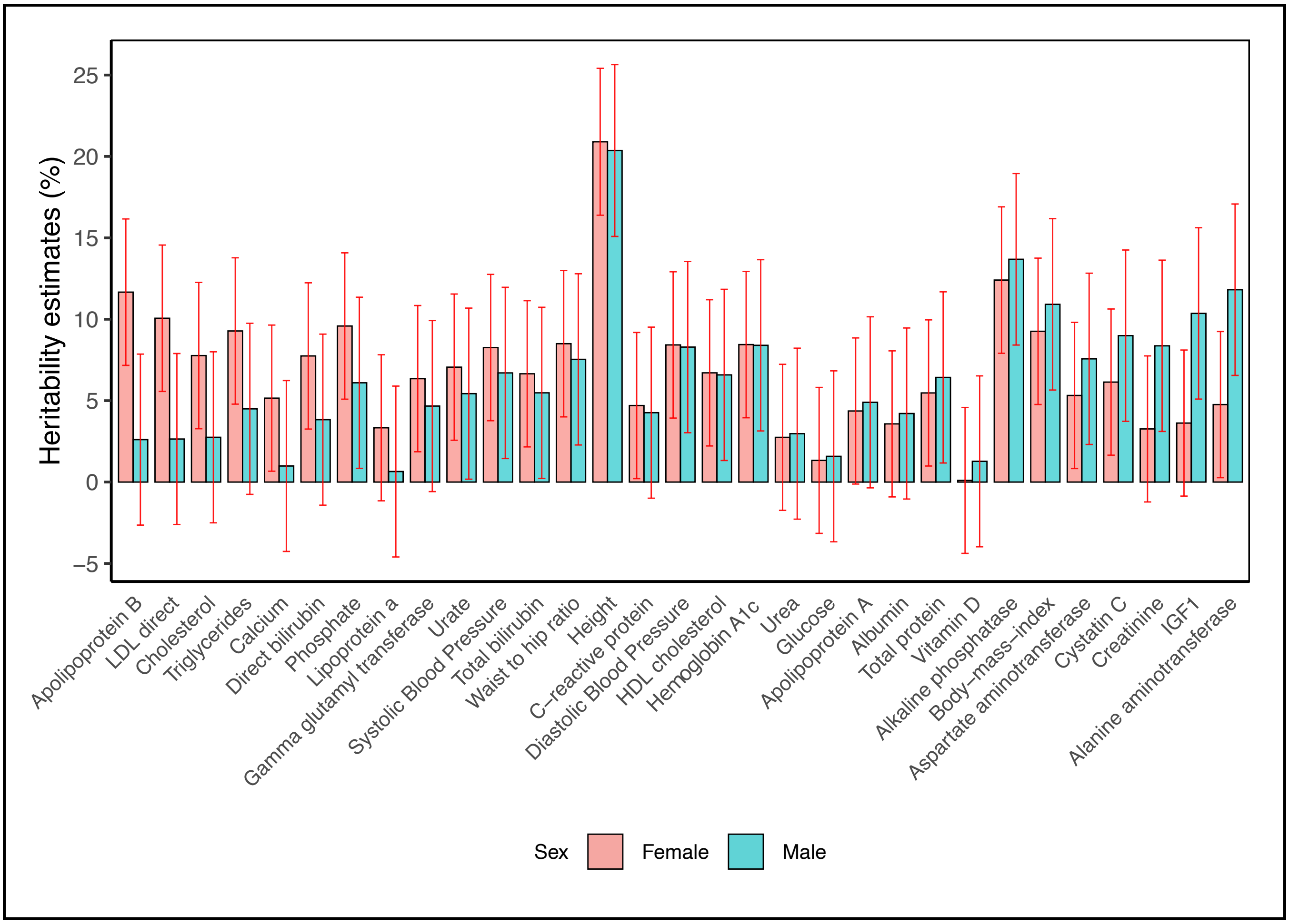

Supplementary Fig.4: Comparison of RV exome-wide heritability estimates between the sexes for 31 continuous traits. Estimation of heritability based on RVs with MAF <0.01 in 92,963 females (light red) and 74,385 males (blue). Red error bar represents 95% confidence intervals of the estimated heritability. Differences in heritability estimates between the sexes appear heterogenous but remains statistically non-significant (p-value > 0.05).

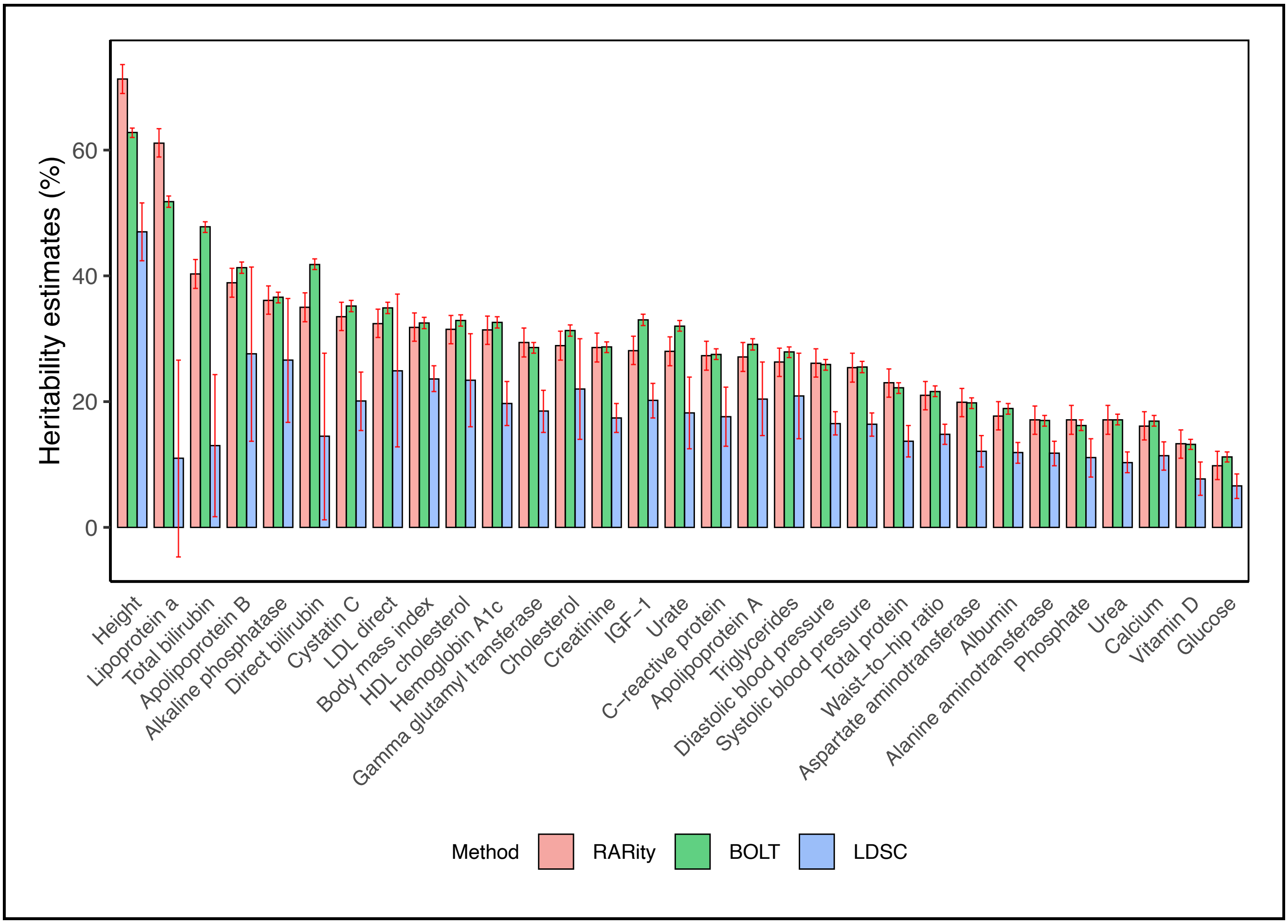

Supplementary Fig.5: Comparison of methods to estimate common variant (MAF>0.01) heritability estimates. The methods under comparison are RARity (light red), BOLT (green), and LDSC (blue) in 31 complex traits, that were adjusted for age, sex and the first 20 PCs. See online Methods for a description of each method. The red error bar represents 95% confidence intervals of the heritability estimates.

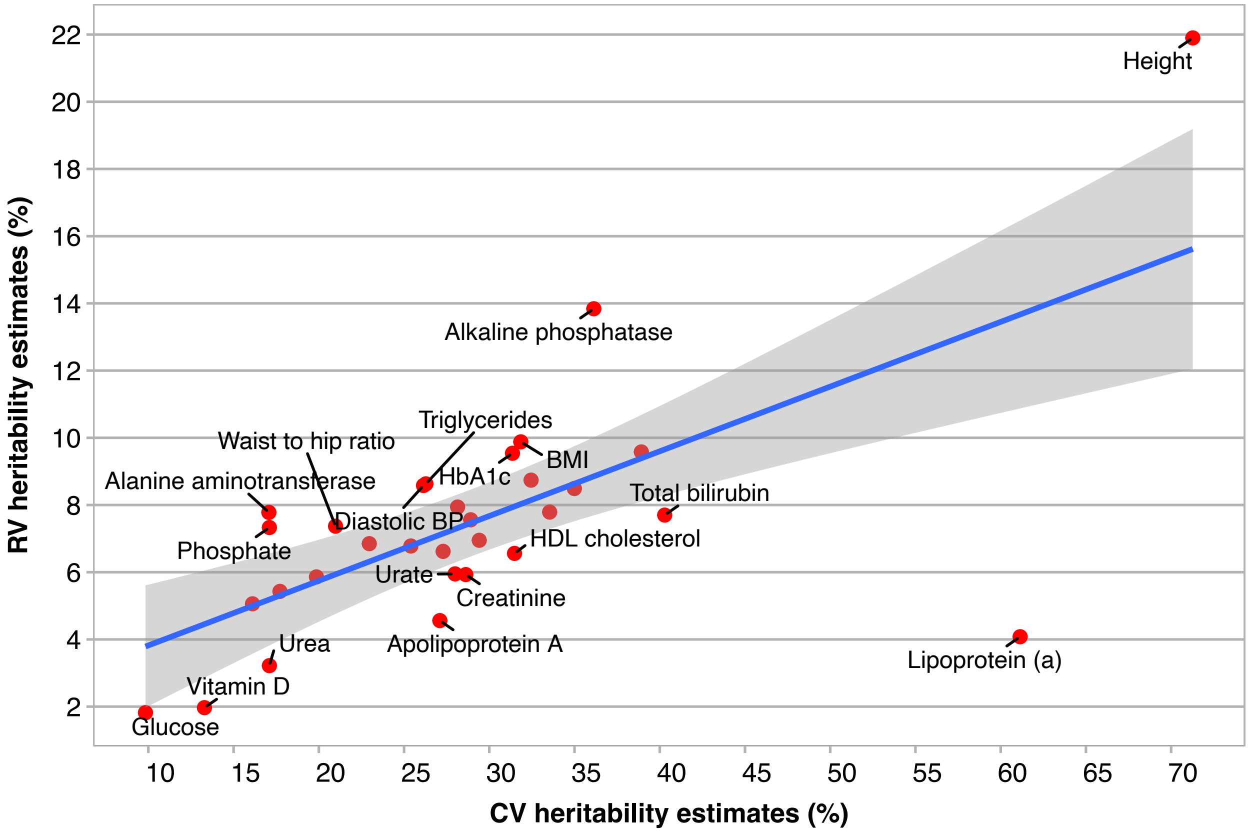

Supplementary Fig.6: Correlation between the contribution of RV and CV to complex traits heritability. Both CV and RV contributions to heritability was estimated with RARity, however the LD pruning threshold was varied (Methods) depending on the variant type. Exome-wide blocks with 5,000 RVs/ block were used to estimate h^2_{RV}, while genome-wide blocks with 20,000 CVs/block were used to estimate h^2_{CV}. Uncertainty in the relationship is expressed as 95% CI, denoted with the grey, shaded area.

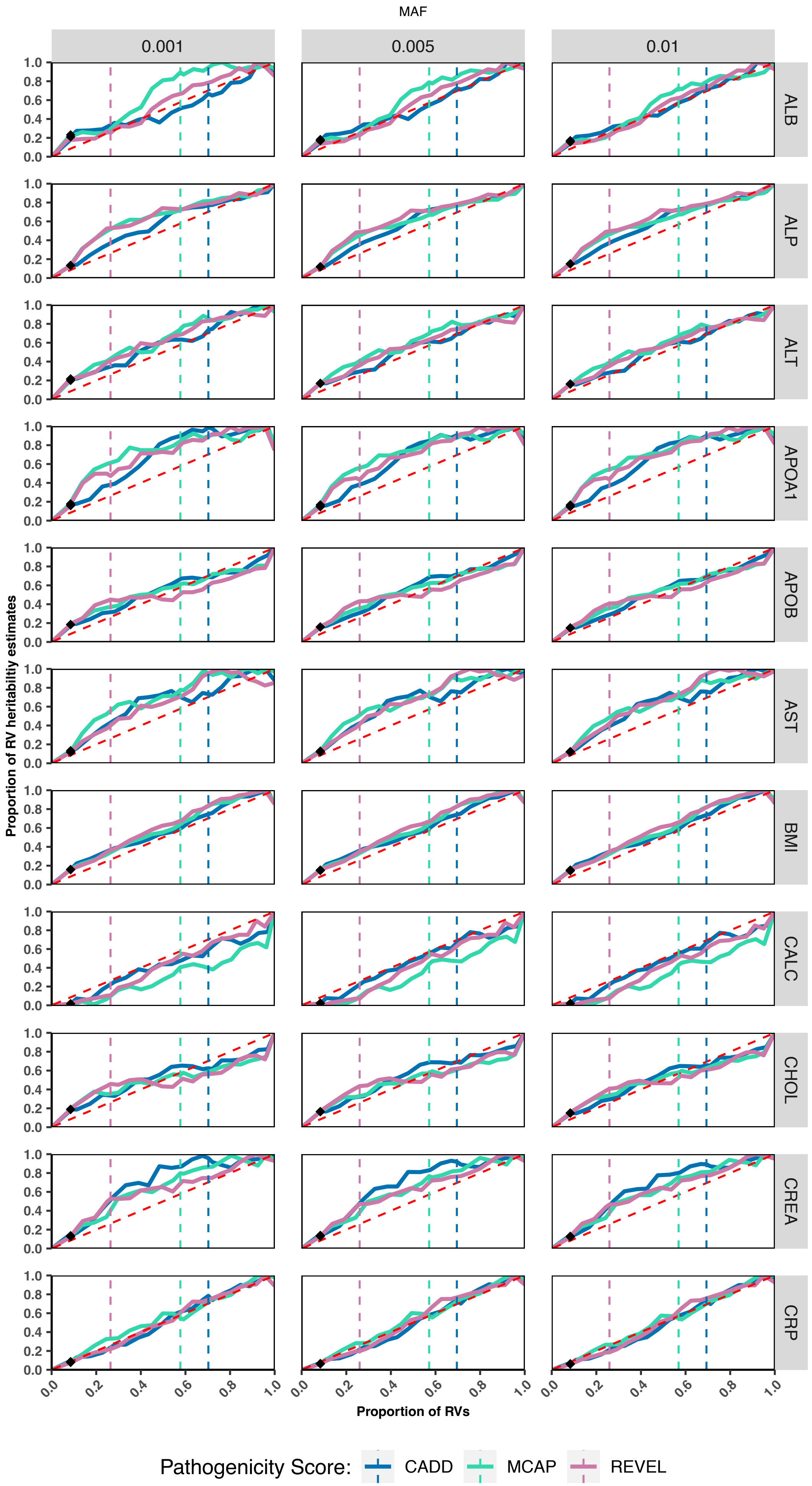
MAF
0.001
0.005
0.01
ALB
ALP
ALT
APOA1
APOB
AST
BMI
CALC
CHOL
CREA
CRP
Proportion of RV heritabiltiy estimates
Proportion of RVs
Pathogenicity Score:
CADD
MCAP
REVEL

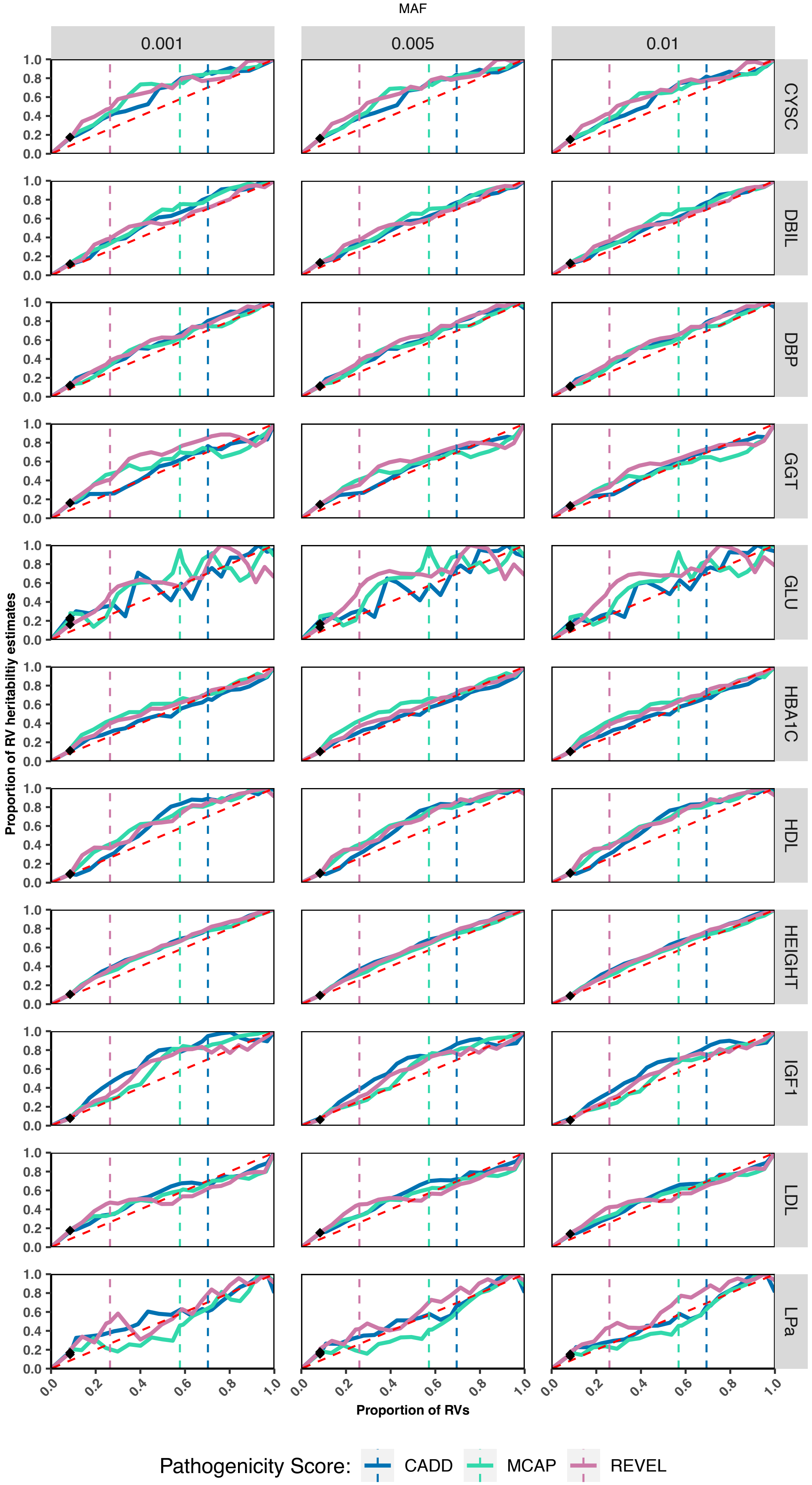
MAF
0.001
0.005
0.01
CYSC
DBIL
DBP
GGT
GLU
HBA1C
HDL
HEIGHT
IGF1
LDL
LPa
Proportion of RV heritability estimates
Proportion of RVs
0.0
0.2
0.4
0.6
0.8
1.0
Pathogenicity Score:
CADD
MCAP
REVEL

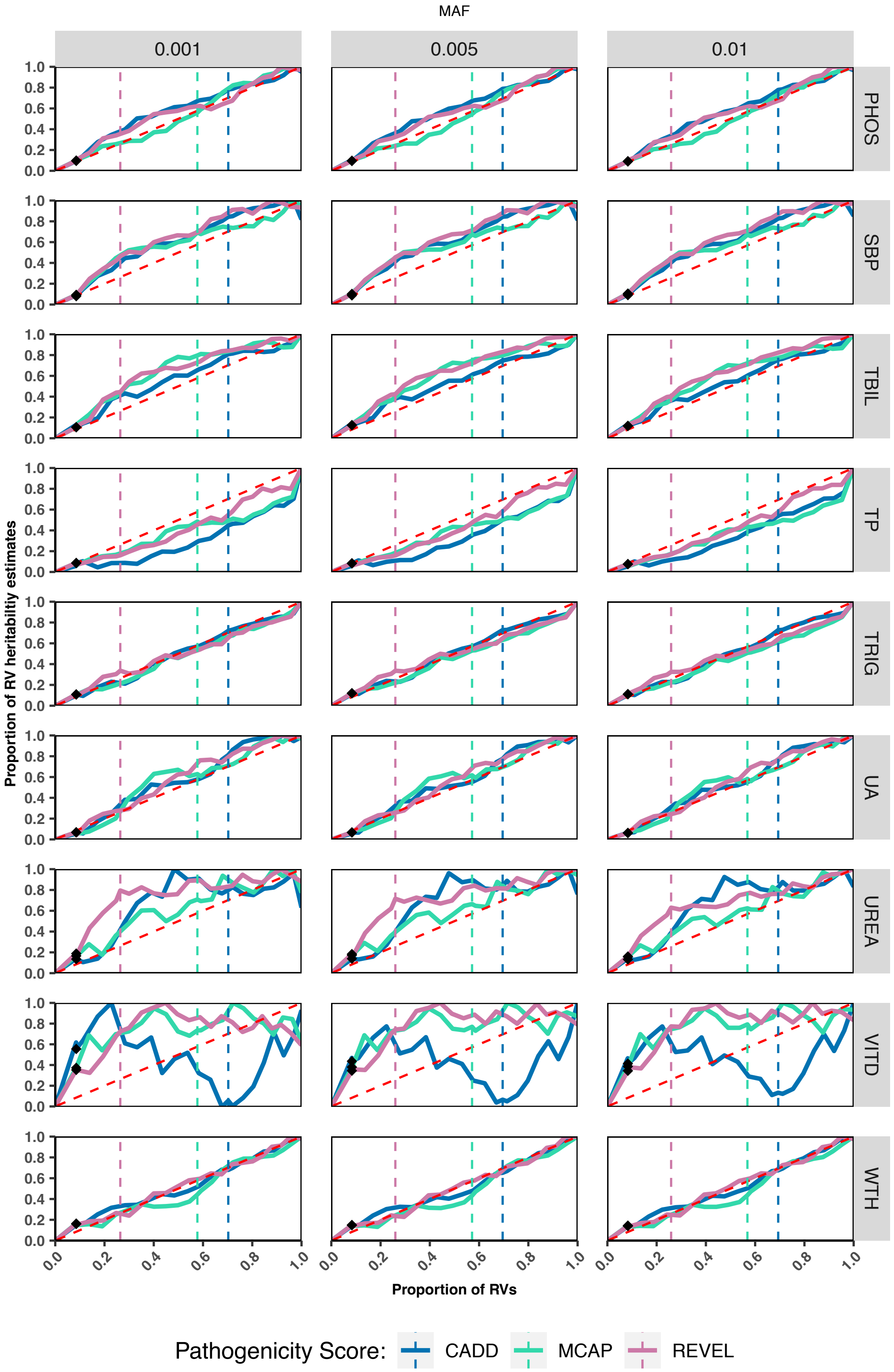

Supplementary Fig.7: Impact of pathogenicity scores on variance explained by RVs for 31 complex traits. Illustration of the proportion of RV heritability explained (*y* axis) as a function of incorporating increasingly "deleterious" genetic variants (*x* axis). Proportion of heritability estimates is the fraction of estimates in relation to all protein altering and LoF variants within the MAF categories. The vertical, dashed lines represent the binary thresholds recommended to define pathogenicity for CADD (dark blue), M-CAP (green) and REVEL (magenta). The black diamond marks the point of last inclusion of LoF variants, which were prioritized before missense mutations. The diagonal dashed red lines represent the scenario wherein RVs uniformly contribute to h^2_{RV}, irrespective of pathogenicity score. Description of the abbreviated traits are available in Supplementary Table 1.

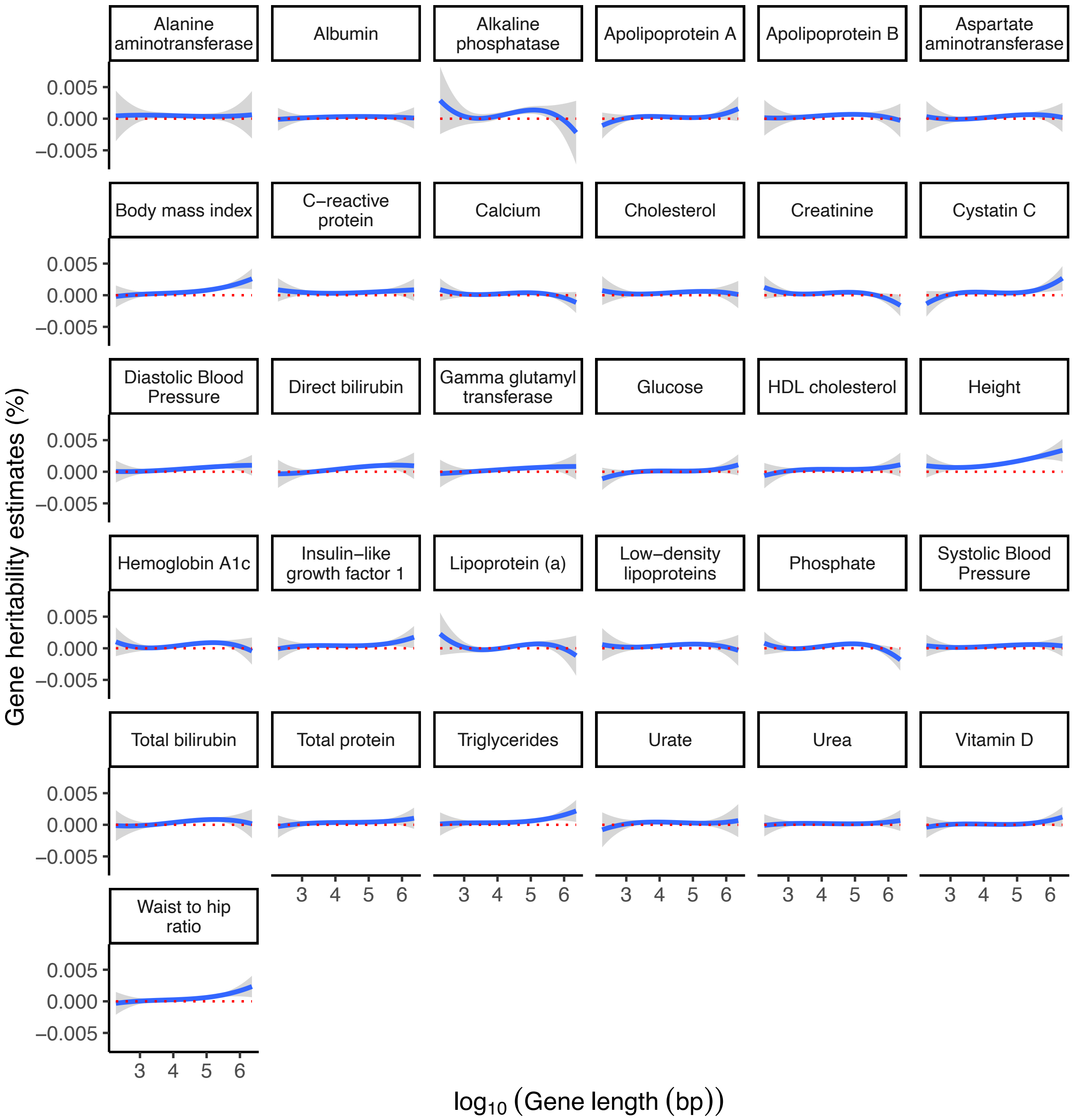

Supplementary Fig.8: Spline plots for the associations of $\log_{10}$ (Gene length (bp)) with $h^2_{\text{gene-RV}}$. Transcripts with the largest length were used as a measure of gene-length. A base spline graph with three degrees of freedom has been used to show the relationship between gene length and $h^2_{\text{gene-RV}}$. Red-dotted lines represent the expected $h^2_{\text{gene-RV}} = 0$, under the null-hypothesis. Uncertainty in the relationship is expressed by a 95% CI (grey band).

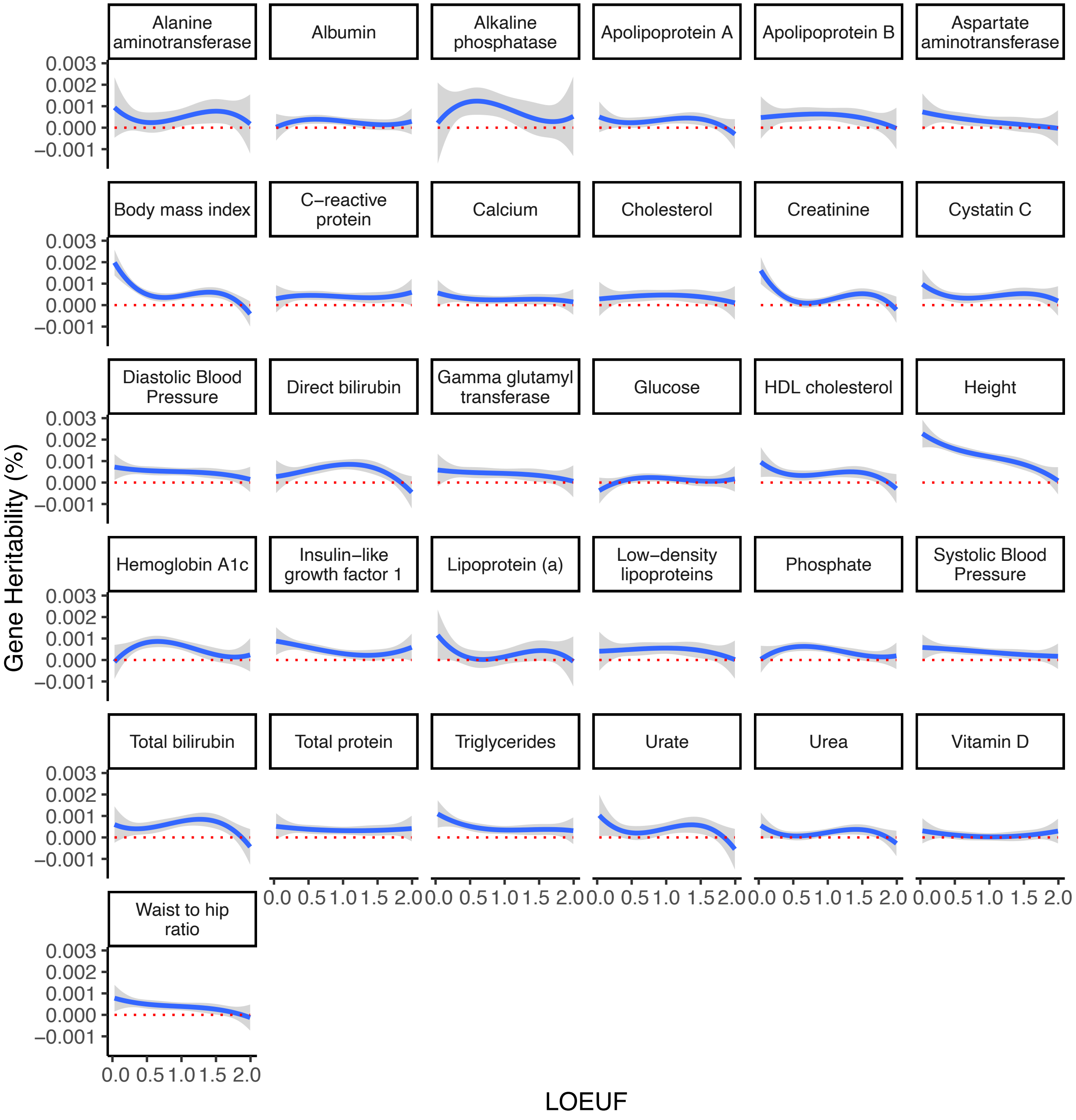

Supplementary Fig.9: Spline plots for the associations of evolutionary constraint (LOEUF) with $h^2_{\text{gene-RV}}$. Relationship between the gene evolutionary constraints and $h^2_{\text{gene-RV}}$ is illustrated using a base spline with three degrees of freedom. Low LOEUF scores indicate strong selection against predicted loss-of-function (pLoF) variation in a given gene, while high LOEUF scores suggest a relatively higher tolerance to inactivation. Red-dotted lines represent the expected $h^2_{\text{gene-RV}} = 0$, under the null-hypothesis. Uncertainty in the relationship is expressed by a 95% CI (grey band).

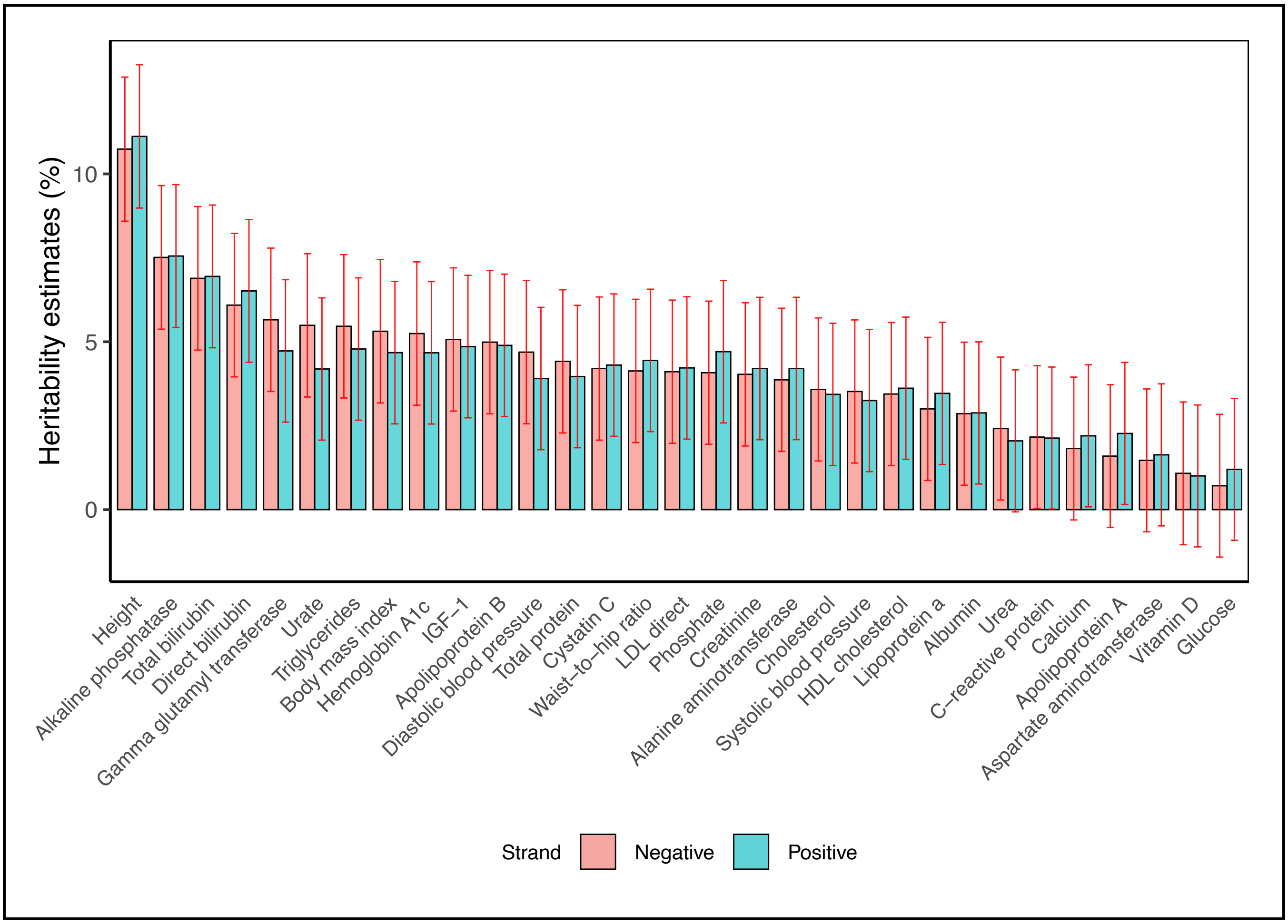

Supplementary Fig.10: Comparison of RV heritability estimates between the genes encoded in the positive vs negative strands. Estimation of trait heritability ($h^2_{\text{RV-gene-tot}}$) +/- 95% CI, was based on RVs with MAF < 0.01, belonging to genes on either the positive strand (blue) or the negative strands (light red). Gene-wise block construct was utilized for the estimation of each $h^2_{\text{RV-gene}}$, followed by the estimation of the $h^2_{\text{RV-gene-tot}}$ as described in methods. Bright-red error bars denote 95% CI of the $h^2_{\text{RV-gene-tot}}$.

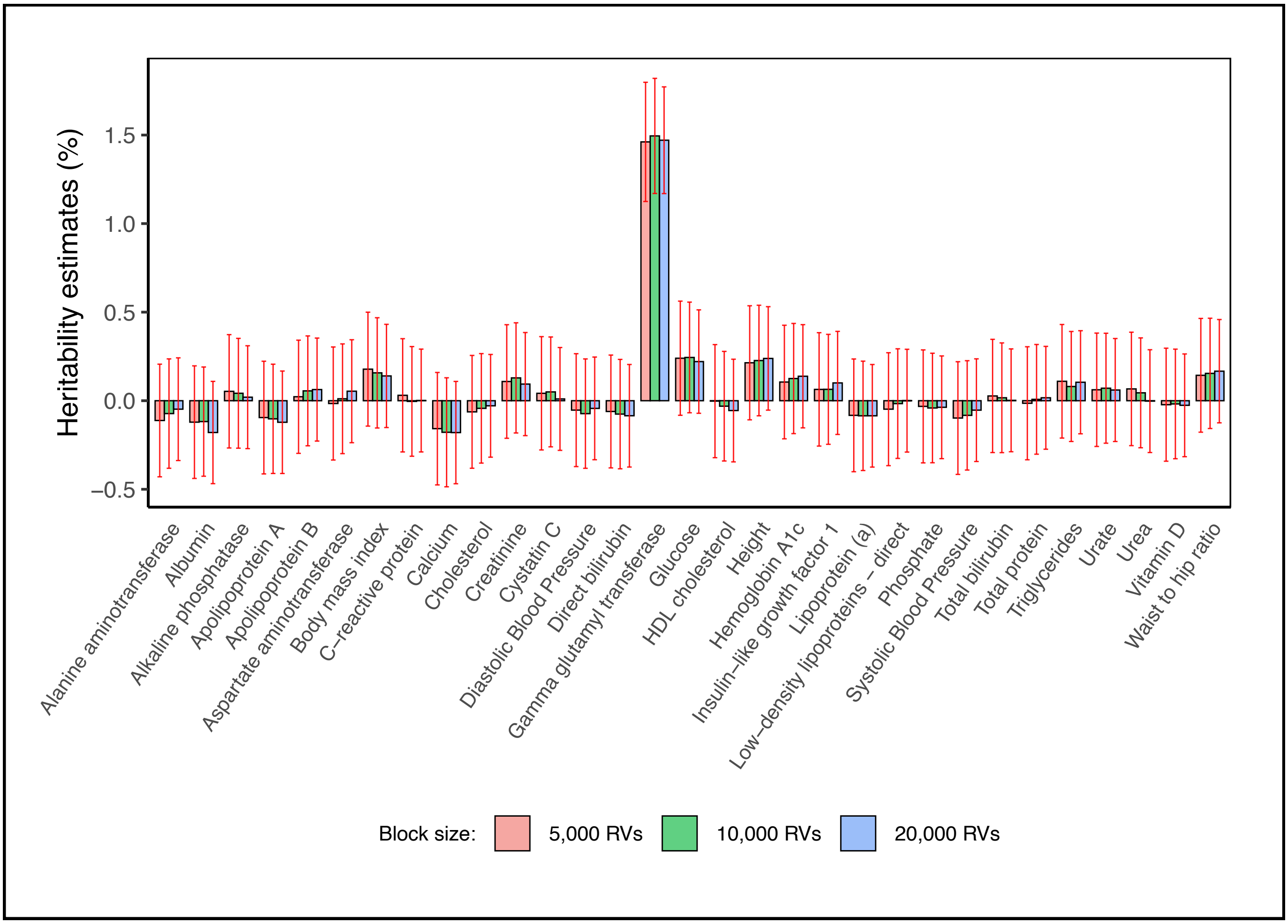

Supplementary Fig.11: Impact of block size on RV heritability estimates. Estimation of heritability (h^2_{RV}) +/- 95% CI, based on protein altering and LoF RVs with MAF < 0.01, using 20,000 variants from chromosome 22, partitioned into blocks of either 5,000 (light red), 10,000 (green) or 20,000 (blue) consecutive RVs. Bright-red error bars denote 95% confidence intervals of the h^2_{RV}.

Supplementary Table 1: Abbreviations used to describe the phenotypes. The list of phenotypes in this study includes 26 biomarkers and 5 anthropomorphic traits in 167,348 unrelated Caucasian participants from the UKB.

Index	TRAITS	ABBREVIATIONS
1	Albumin	ALB
2	Alkaline phosphatase	ALP
3	Alanine aminotransferase	ALT
4	Apolipoprotein A	APOA1
5	Apolipoprotein B	APOB
6	Aspartate aminotransferase	AST
7	Calcium	CALC
8	Cholesterol	CHOL
9	Creatinine	CREA
10	C-reactive protein	CRP
11	Cystatin C	CYSC
12	Direct bilirubin	DBIL
13	Gamma glutamyl transferase	GGT
14	Glucose	GLU
15	Hemoglobin A1c	HBA1C
16	HDL cholesterol	HDL
17	Insulin-like growth factor 1	IGF1
18	Low-density lipoproteins - direct	LDL
19	Lipoprotein (a)	LPa
20	Phosphate	PHOS
21	Total bilirubin	TBIL
22	Total protein	TP
23	Triglycerides	TRIG
24	Urea	UREA
25	Urate	UA
26	Vitamin D	VITD
27	Height	HIGHT
28	Waist to hip ratio	WTH
29	Systolic Blood Pressure	SBP
30	Diastolic Blood Pressure	DBP
31	Body mass index	BMI

Supplementary Table 2: Medications used for adjusting biomarkers values. Field ids and the medication code used for glucose lowering, cholesterol lowering and hypertension medications. Adjustment for these medications is further described in methods.

**Glucose lowering medication**		**Cholesterol lowering medications**		**Hypertension medications**	
**Field 20003 code**	**Description**	**Field 20003 code**	**Description**	**Field 6177 code**	**Description**
1140868902	acarbose	1141146234	atorvastatin	2	Blood pressure medication
1140857584	acetohexamide	1141192414	crestor 10mg tablet		
1141171652	actos 15mg tablet	1141192736	ezetimibe	**Field 6153 code**	**Description**
1140868866	bromocriptine	1140888594	fluvastatin	2	Blood pressure medication
1140874706	chlorpropamide	1141146138	lipitor 10mg tablet		
1140874746	diamicron 80mg tablet	1140888648	pravastatin		
1140866568	disopyramide	1141192410	rosuvastatin		
1141157186	disopyramide product	1140861958	simvastatin		
1140857518	eudemine 50mg tablet	1140881748	zocor 10mg tablet		
1140874718	glibenclamide	1141200040	zocor heart-pro 10mg tablet		
1140857494	glibornuride	1140864592	lescol 20mg capsule		
1140874744	gliclazide				
1141152590	glimepiride	**Field 6177 code**	**Description**		
1140874646	glipizide	1	Cholesterol lowering medications		
1141157284	glipizide product	**Field 6153 code**	**Description**		
1140874658	gliquidone	1	Cholesterol lowering medications		
1140874686	glucophage 500mg tablet				
1140857500	glymidine				
1140874754	guar gum				
1140884600	metformin				
1140869112	mifepristone				
1141157302	mifepristone product				
1140874652	minodiab 2.5mg tablet=glipizide				
1141173882	nateglinide				
1140884338	pentamidine				
1141171646	pioglitazone				
1140874420	quinine				
1141168660	repaglinide				
1141177600	rosiglitazone				
1141189090	rosiglitazone 1mg / metformin 500mg tablet				
1141173786	starlix 60mg tablet=nateglinide				
1141182110	sulfadiazine				
1140874664	tolazamide				
1140874674	tolbutamide				
1141153254	troglitazone				
**Field 6177 code**	**Description**				
3	Insulin				
**Field 6153 code**	**Description**				
3	Insulin				

Supplementary Table 3: Comparison of RV heritability estimates derived using gene-burden, gene-wise and exome-wide blocks. $h^2_{RV\text{-}burden}$ = RV heritability estimates derived using gene-burden block construct, $h^2_{RV\text{-}gene\text{-}tot}$ = RV heritability estimates derived using gene block construct, h^2_{RV} = RV heritability estimates derived using exome-wide block construct with ~5000 consecutive variants in each block. LCL= lower confidence level, UCL=upper confidence level. Comparison shows percentage difference in trait heritability between the methods.

	**Gene Burden**			**Gene-wise**			**Exome-wide**			**Comparison of RV heritability**		
**Trait**	$h^2_{RV\text{-}burden}$	**LCL**	**UCL**	$h^2_{RV\text{-}gene\text{-}tot}$	**LCL**	**UCL**	h^2_{RV}	**LCL**	**UCL**	**Exome-wise vs Gene burden**	**Gene-wise vs Gene burden**	**Gene-wise vs Exome-wise**
ALB	0.0099	0.0070	0.0128	0.0554	0.0258	0.0849	0.0543	0.0257	0.0829	82%	82%	2%
ALP	0.0438	0.0406	0.0469	0.1426	0.1129	0.1723	0.1384	0.1097	0.1671	68%	69%	3%
ALT	0.0161	0.0132	0.0191	0.0795	0.0499	0.1091	0.0778	0.0492	0.1064	79%	80%	2%
APOA1	0.0129	0.0100	0.0158	0.0473	0.0177	0.0768	0.0456	0.0170	0.0742	72%	73%	4%
APOB	0.0151	0.0122	0.0180	0.0970	0.0674	0.1266	0.0958	0.0672	0.1245	84%	84%	1%
AST	0.0101	0.0072	0.0130	0.0557	0.0261	0.0853	0.0586	0.0301	0.0872	83%	82%	-5%
CALC	0.0065	0.0036	0.0093	0.0485	0.0189	0.0781	0.0506	0.0221	0.0792	87%	87%	-4%
CHOL	0.0137	0.0108	0.0166	0.0740	0.0444	0.1036	0.0756	0.0470	0.1042	82%	81%	-2%
CREA	0.0130	0.0101	0.0159	0.0612	0.0317	0.0908	0.0593	0.0308	0.0879	78%	79%	3%
CRP	0.0142	0.0113	0.0171	0.0714	0.0418	0.1009	0.0662	0.0376	0.0948	79%	80%	7%
CYSC	0.0144	0.0115	0.0173	0.0778	0.0482	0.1074	0.0779	0.0493	0.1065	81%	81%	0%
DBIL	0.0100	0.0071	0.0129	0.0992	0.0695	0.1288	0.0849	0.0563	0.1135	88%	90%	14%
GGT	0.0100	0.0071	0.0129	0.0751	0.0455	0.1047	0.0695	0.0409	0.0981	86%	87%	7%
GLU	0.0056	0.0027	0.0084	0.0192	-0.0103	0.0487	0.0182	-0.0103	0.0467	69%	71%	5%
HBA1C	0.0104	0.0075	0.0133	0.0960	0.0663	0.1256	0.0954	0.0667	0.1240	89%	89%	1%
HDL	0.0140	0.0111	0.0169	0.0675	0.0379	0.0971	0.0656	0.0370	0.0942	79%	79%	3%
IGF1	0.0116	0.0087	0.0145	0.0802	0.0506	0.1098	0.0794	0.0508	0.1081	85%	86%	1%
LDL	0.0138	0.0109	0.0167	0.0865	0.0569	0.1161	0.0874	0.0588	0.1160	84%	84%	-1%
LPa	0.0128	0.0099	0.0157	0.0427	0.0132	0.0723	0.0408	0.0123	0.0694	69%	70%	4%
PHOS	0.0107	0.0078	0.0136	0.0762	0.0466	0.1058	0.0733	0.0446	0.1019	85%	86%	4%
TBIL	0.0103	0.0074	0.0131	0.0930	0.0633	0.1226	0.0770	0.0484	0.1056	87%	89%	17%
TP	0.0093	0.0064	0.0122	0.0690	0.0394	0.0986	0.0685	0.0399	0.0971	86%	86%	1%
TRIG	0.0108	0.0079	0.0137	0.0839	0.0543	0.1135	0.0863	0.0577	0.1149	88%	87%	-3%
UREA	0.0044	0.0015	0.0072	0.0343	0.0048	0.0639	0.0322	0.0037	0.0608	87%	87%	6%
UA	0.0191	0.0162	0.0221	0.0634	0.0338	0.0930	0.0595	0.0309	0.0881	68%	70%	6%
VITD	0.0044	0.0016	0.0073	0.0198	-0.0097	0.0493	0.0197	-0.0088	0.0482	77%	78%	0%
SBP	0.0060	0.0031	0.0089	0.0662	0.0366	0.0958	0.0678	0.0392	0.0964	91%	91%	-2%
DBP	0.0073	0.0044	0.0101	0.0872	0.0576	0.1168	0.0858	0.0572	0.1144	92%	92%	2%
BMI	0.0076	0.0047	0.0104	0.0990	0.0693	0.1286	0.0988	0.0702	0.1275	92%	92%	0%
WTH	0.0050	0.0022	0.0079	0.0736	0.0440	0.1032	0.0737	0.0451	0.1023	93%	93%	0%
HEIGHT	0.0248	0.0218	0.0278	0.2231	0.1932	0.2529	0.2190	0.1902	0.2478	89%	89%	2%

Supplementary Table 4: RV heritability by MAF bins calculated using gene-wise blocks. N variants= number of variants present in each MAF bin. LCL= lower confidence level, UCL=upper confidence level.

**MAF**	**0.01 to 0.005**				**0.005 to 0.001**				**0.01 to 0.001**				**< 0.001**			
**Trait**	**N variants**	$h^2_{RV\text{-}gene\text{-}tot}$	**LCL**	**UCL**	**N variants**	$\mathbf{h^2_{RV\text{-}gene\text{-}tot}}$	**LCL**	**UCL**	**N variants**	$h^2_{RV\text{-}gene\text{-}tot}$	**LCL**	**UCL**	**N variants**	$h^2_{RV\text{-}gene\text{-}tot}$	**LCL**	**UCL**
ALB	6509	0.0063	0.0043	0.0084	23714	0.0093	0.0056	0.0131	29552	0.0146	0.0104	0.0188	1514538	0.0355	0.0066	0.0643
ALP	6509	0.0096	0.0075	0.0117	23714	0.0247	0.0208	0.0286	29552	0.0332	0.0288	0.0376	1514538	0.1110	0.0820	0.1400
ALT	6509	0.0040	0.0021	0.0060	23714	0.0136	0.0099	0.0174	29552	0.0177	0.0135	0.0220	1514538	0.0539	0.0250	0.0827
APOA1	6509	0.0049	0.0029	0.0069	23714	0.0143	0.0105	0.0181	29552	0.0181	0.0138	0.0223	1514538	0.0327	0.0038	0.0615
APOB	6509	0.0139	0.0117	0.0161	23714	0.0180	0.0142	0.0218	29552	0.0299	0.0256	0.0343	1514538	0.0710	0.0421	0.0999
AST	6509	0.0060	0.0040	0.0080	23714	0.0093	0.0055	0.0130	29552	0.0146	0.0104	0.0188	1514538	0.0398	0.0109	0.0686
CALC	6509	0.0040	0.0020	0.0060	23714	0.0077	0.0040	0.0114	29552	0.0120	0.0079	0.0162	1514538	0.0367	0.0079	0.0656
CHOL	6509	0.0101	0.0080	0.0122	23714	0.0146	0.0108	0.0184	29552	0.0232	0.0189	0.0275	1514538	0.0541	0.0253	0.0830
CREA	6509	0.0067	0.0047	0.0088	23714	0.0136	0.0098	0.0173	29552	0.0195	0.0152	0.0237	1514538	0.0450	0.0162	0.0739
CRP	6509	0.0062	0.0041	0.0082	23714	0.0134	0.0096	0.0171	29552	0.0188	0.0145	0.0230	1514538	0.0542	0.0253	0.0831
CYSC	6509	0.0064	0.0043	0.0084	23714	0.0128	0.0091	0.0166	29552	0.0177	0.0135	0.0219	1514538	0.0620	0.0331	0.0909
DBIL	6509	0.0084	0.0063	0.0105	23714	0.0094	0.0056	0.0131	29552	0.0171	0.0129	0.0213	1514538	0.0766	0.0476	0.1055
GGT	6509	0.0093	0.0072	0.0114	23714	0.0124	0.0086	0.0161	29552	0.0207	0.0165	0.0250	1514538	0.0491	0.0203	0.0780
GLU	6509	0.0032	0.0013	0.0052	23714	0.0046	0.0010	0.0083	29552	0.0079	0.0038	0.0120	1514538	0.0138	-0.0151	0.0426
HBA1C	6509	0.0099	0.0078	0.0121	23714	0.0164	0.0126	0.0202	29552	0.0258	0.0215	0.0301	1514538	0.0772	0.0483	0.1061
HDL	6509	0.0048	0.0028	0.0068	23714	0.0146	0.0108	0.0184	29552	0.0185	0.0143	0.0227	1514538	0.0515	0.0226	0.0804
IGF1	6509	0.0071	0.0050	0.0091	23714	0.0161	0.0123	0.0199	29552	0.0222	0.0179	0.0264	1514538	0.0542	0.0253	0.0831
LDL	6509	0.0113	0.0091	0.0134	23714	0.0151	0.0113	0.0189	29552	0.0245	0.0202	0.0288	1514538	0.0644	0.0355	0.0933
LPa	6509	0.0066	0.0045	0.0086	23714	0.0177	0.0138	0.0215	29552	0.0230	0.0187	0.0272	1514538	0.0235	-0.0053	0.0524
PHOS	6509	0.0078	0.0057	0.0099	23714	0.0113	0.0076	0.0151	29552	0.0180	0.0138	0.0223	1514538	0.0618	0.0329	0.0907
TBIL	6509	0.0095	0.0074	0.0116	23714	0.0112	0.0074	0.0149	29552	0.0202	0.0159	0.0244	1514538	0.0686	0.0397	0.0975
TP	6509	0.0091	0.0070	0.0112	23714	0.0099	0.0061	0.0136	29552	0.0179	0.0137	0.0221	1514538	0.0503	0.0214	0.0791
TRIG	6509	0.0073	0.0052	0.0094	23714	0.0116	0.0078	0.0153	29552	0.0182	0.0140	0.0224	1514538	0.0629	0.0340	0.0918
UREA	6509	0.0045	0.0025	0.0065	23714	0.0042	0.0005	0.0078	29552	0.0086	0.0045	0.0128	1514538	0.0197	-0.0091	0.0486
UA	6509	0.0059	0.0039	0.0079	23714	0.0099	0.0061	0.0136	29552	0.0151	0.0110	0.0193	1514538	0.0490	0.0202	0.0779
VITD	6509	0.0018	-0.0002	0.0037	23714	0.0066	0.0029	0.0103	29552	0.0083	0.0042	0.0125	1514538	0.0120	-0.0168	0.0409
SBP	6509	0.0053	0.0033	0.0073	23714	0.0089	0.0052	0.0126	29552	0.0135	0.0093	0.0177	1514538	0.0549	0.0260	0.0838
DBP	6509	0.0060	0.0040	0.0080	23714	0.0082	0.0045	0.0119	29552	0.0136	0.0094	0.0178	1514538	0.0783	0.0494	0.1072
BMI	6509	0.0054	0.0034	0.0074	23714	0.0089	0.0052	0.0127	29552	0.0141	0.0099	0.0183	1514538	0.0869	0.0580	0.1158
WTH	6509	0.0051	0.0030	0.0071	23714	0.0073	0.0036	0.0110	29552	0.0120	0.0079	0.0162	1514538	0.0647	0.0358	0.0936
HEIGHT	6509	0.0171	0.0148	0.0193	23714	0.0301	0.0262	0.0341	29552	0.0460	0.0415	0.0506	1514538	0.1726	0.1435	0.2016

Supplementary Table 5: Rare coding variant heritability as a function of log_{10}(Gene-length). *P-values* were calculated using a multivariable linear regression model, with log_{10} (gene-length) as the independent predictor and RV gene-heritability estimates as the outcome variable, adjusted for sex and the first 20 principal components of ancestry.

Trait	Estimate	Standard error	R^2	*P-value*
HEIGHT	7.68×10^{-2}	1.16×10^{-2}	2.41×10^{-3}	3.34×10^{-11}
BMI	5.40×10^{-2}	1.16×10^{-2}	1.19×10^{-3}	3.17×10^{-6}
DBIL	4.72×10^{-2}	1.16×10^{-2}	9.11×10^{-4}	4.62×10^{-5}
WTH	4.55×10^{-2}	1.16×10^{-2}	8.47×10^{-4}	8.58×10^{-5}
DBP	4.20×10^{-2}	1.16×10^{-2}	7.21×10^{-4}	2.91×10^{-4}
TBIL	3.65×10^{-2}	1.16×10^{-2}	5.45×10^{-4}	1.63×10^{-3}
HBA1C	3.58×10^{-2}	1.16×10^{-2}	5.24×10^{-4}	2.00×10^{-3}
GGT	3.19×10^{-2}	1.16×10^{-2}	4.17×10^{-4}	5.87×10^{-3}
TRIG	3.10×10^{-2}	1.16×10^{-2}	3.92×10^{-4}	7.54×10^{-3}
PHOS	3.09×10^{-2}	1.16×10^{-2}	3.92×10^{-4}	7.56×10^{-3}
AST	2.97×10^{-2}	1.16×10^{-2}	3.61×10^{-4}	1.04×10^{-2}
SBP	2.65×10^{-2}	1.16×10^{-2}	2.87×10^{-4}	2.23×10^{-2}
Lpa	2.38×10^{-2}	1.16×10^{-2}	2.31×10^{-4}	4.01×10^{-2}
ALP	2.14×10^{-2}	1.16×10^{-2}	1.88×10^{-4}	6.46×10^{-2}
CHOL	1.82×10^{-2}	1.16×10^{-2}	1.36×10^{-4}	1.16×10^{-1}
LDL	1.68×10^{-2}	1.16×10^{-2}	1.15×10^{-4}	1.48×10^{-1}
GLU	1.45×10^{-2}	1.16×10^{-2}	8.65×10^{-5}	2.10×10^{-1}
TP	1.44×10^{-2}	1.16×10^{-2}	8.44×10^{-5}	2.15×10^{-1}
APOB	1.42×10^{-2}	1.16×10^{-2}	8.29×10^{-5}	2.19×10^{-1}
CYSC	1.33×10^{-2}	1.16×10^{-2}	7.24×10^{-5}	2.51×10^{-1}
IGF1	1.19×10^{-2}	1.16×10^{-2}	5.84×10^{-5}	3.03×10^{-1}
CRP	1.08×10^{-2}	1.16×10^{-2}	4.75×10^{-5}	3.52×10^{-1}
HDL	9.66×10^{-3}	1.16×10^{-2}	3.82×10^{-5}	4.04×10^{-1}
CALC	8.28×10^{-3}	1.16×10^{-2}	2.80×10^{-5}	4.75×10^{-1}
ALB	7.65×10^{-3}	1.16×10^{-2}	2.39×10^{-5}	5.09×10^{-1}
VITD	6.48×10^{-3}	1.16×10^{-2}	1.72×10^{-5}	5.76×10^{-1}
TEST	-6.41×10^{-3}	1.16×10^{-2}	1.68×10^{-5}	5.80×10^{-1}
ALT	-4.93×10^{-3}	1.16×10^{-2}	9.94×10^{-6}	6.70×10^{-1}
UA	-1.55×10^{-3}	1.16×10^{-2}	9.86×10^{-7}	8.93×10^{-1}
CREA	1.54×10^{-3}	1.16×10^{-2}	9.73×10^{-7}	8.94×10^{-1}
APOA1	9.02×10^{-4}	1.16×10^{-2}	3.33×10^{-7}	9.38×10^{-1}
UREA	-3.36×10^{-4}	1.16×10^{-2}	4.62×10^{-8}	9.77×10^{-1}

Supplementary Table 6: Rare coding variant heritability as a function of evolutionary constraint. *P-values* were calculated using a multivariable linear regression model, with gene evolutionary constraint (LOEUF score) as the independent predictor and RV gene-heritability estimates as the outcome variable, adjusted for sex and the first 20 principal components of ancestry.

Trait	Estimate	Standard error	R^2	*P-value*
HEIGHT	-8.77×10^{-6}	1.30×10^{-6}	2.68×10^{-3}	1.71×10^{-11}
BMI	-4.29×10^{-6}	1.20×10^{-6}	7.52×10^{-4}	3.66×10^{-4}
WTH	-3.24×10^{-6}	1.25×10^{-6}	4.00×10^{-4}	9.37×10^{-3}
IGF1	-2.71×10^{-6}	1.30×10^{-6}	2.56×10^{-4}	3.77×10^{-2}
AST	-3.48×10^{-6}	1.73×10^{-6}	2.40×10^{-4}	4.40×10^{-2}
TRIG	-2.46×10^{-6}	1.28×10^{-6}	2.20×10^{-4}	5.41×10^{-2}
SBP	-2.29×10^{-6}	1.22×10^{-6}	2.10×10^{-4}	5.96×10^{-2}
HBA1C	-2.83×10^{-6}	1.59×10^{-6}	1.86×10^{-4}	7.60×10^{-2}
DBP	-2.01×10^{-6}	1.21×10^{-6}	1.64×10^{-4}	9.60×10^{-2}
PHOS	-1.98×10^{-6}	1.26×10^{-6}	1.46×10^{-4}	1.16×10^{-1}
CREA	-1.61×10^{-6}	1.26×10^{-6}	9.75×10^{-5}	2.00×10^{-1}
HDL	-1.78×10^{-6}	1.40×10^{-6}	9.54×10^{-5}	2.04×10^{-1}
GGT	-1.92×10^{-6}	1.52×10^{-6}	9.47×10^{-5}	2.06×10^{-1}
ALP	-4.53×10^{-6}	3.80×10^{-6}	8.42×10^{-5}	2.33×10^{-1}
APOB	-1.87×10^{-6}	1.99×10^{-6}	5.28×10^{-5}	3.45×10^{-1}
CALC	-9.30×10^{-7}	1.23×10^{-6}	3.39×10^{-5}	4.49×10^{-1}
DBIL	-1.13×10^{-6}	1.54×10^{-6}	3.17×10^{-5}	4.64×10^{-1}
ALB	-9.06×10^{-7}	1.25×10^{-6}	3.10×10^{-5}	4.69×10^{-1}
LDL	-1.09×10^{-6}	1.82×10^{-6}	2.11×10^{-5}	5.51×10^{-1}
ALT	1.67×10^{-6}	2.83×10^{-6}	2.05×10^{-5}	5.56×10^{-1}
UA	-1.10×10^{-6}	1.95×10^{-6}	1.88×10^{-5}	5.73×10^{-1}
GLU	5.13×10^{-7}	1.22×10^{-6}	1.05×10^{-5}	6.74×10^{-1}
TP	-5.10×10^{-7}	1.23×10^{-6}	1.01×10^{-5}	6.79×10^{-1}
APOA1	-5.57×10^{-7}	1.44×10^{-6}	8.88×10^{-6}	6.99×10^{-1}
CYSC	-5.08×10^{-7}	1.41×10^{-6}	7.66×10^{-6}	7.19×10^{-1}
CHOL	-5.09×10^{-7}	1.61×10^{-6}	5.94×10^{-6}	7.51×10^{-1}
VITD	-1.81×10^{-7}	1.18×10^{-6}	1.39×10^{-6}	8.78×10^{-1}
LPa	-3.53×10^{-7}	2.39×10^{-6}	1.30×10^{-6}	8.82×10^{-1}
TBIL	-1.60×10^{-7}	1.70×10^{-6}	5.24×10^{-7}	9.25×10^{-1}
UREA	1.07×10^{-7}	1.22×10^{-6}	4.58×10^{-7}	9.30×10^{-1}
CRP	-2.04×10^{-8}	1.29×10^{-6}	1.49×10^{-8}	9.87×10^{-1}

Supplementary Table 7: Heritability of height originating from RVs in selected gene clusters. LCL= lower confidence level, UCL=upper confidence level.

Cluster	Number of genes	Number of variants	RV heritability	LCL	UCL
Hemoglobin	10	159	0.000160	-0.000159	0.000479
Histone	77	2099	0.000415	-0.000675	0.00150
HOX	226	14735	0.00118	-0.001679	0.00404
Olfactory	366	18366	0.00315	-0.0000697	0.00637
Protocadherin	59	7034	-0.00124	-0.00317	0.000690

LCL= lower confidence limit, UCL=upper confidence limit
