# Supplementary material for: A method to estimate the contribution of rare coding variants to complex trait heritability
Source: Nat Commun. 2024 Feb 9;15:1245. doi: 10.1038/s41467-024-45407-8 (PMC10858280; doi:10.1038/s41467-024-45407-8)
Supplement: Supplementary file 3 — Description of Additional Supplementary Files [file 41467_2024_45407_MOESM3_ESM.pdf]

Description of Additional Supplementary Files

File Name: Supplementary Data 1
Description: Heritability estimates from twin and pedigree studies. In this table, selected reports of pedigree/twin-based trait heritability are presented for comparison with RARity based heritability estimates from combined rare and common variants (h^2_{tot}). Rows highlighted in green indicate the recovery of pedigree/twin-based heritability estimates through RARity, as these estimates lie within the range historically reported in the referenced literature.

File Name: Supplementary Data 2
Description: Genes contributing significantly to the rare coding variant heritability estimates of complex traits. RARity was used to determine the heritability estimates for all genes with qualifying RVs <MAF 0.01. This table shows the significant genes passing the Bonferroni's *p-value* significance threshold corrected for 18,214 genes ($h^2_{\text{RV-gene}}$ p-value $< 2.75 \times 10^{-6}$), where the $h^2_{\text{RV-gene}}$ p-values were derived using F-test. 152 genes had significant $h^2_{RV\text{-}gene}$ for one or more traits, representing 218 distinct gene-biomarker relationships.

File Name: Supplementary Data 3
Description: Association between the genes contributing significantly to RV heritability and diseases based on DisGeNET platform. The genes with significant contributions to trait heritability, as identified through RARity, were examined for disease associations using the disgenet2r package from the DisGeNET platform. Among the 152 genes with significant $h^2_{\text{RV-gene}}$, 115 genes are linked to multiple diseases, as presented in this table. The data is specifically filtered to include only literature-supported connections (GDA Score > = 0.3).

File Name: Supplementary Data 4
Description: List of druggable and clinically actionable genes retrieved from Drug gene interaction database (DGIdb). RARIty was utilized to identify the genes contributing significantly towards trait heritability, and subsequently the DGIdb (v4.2.0) database was employed to categorize the genes as "clinically actionable" or part of the "druggable genome." The table presents the genes that fall into either the clinically actionable or druggable categories.

File Name: Supplementary Data 5
Description: Functional annotation of the genes contributing significantly to trait heritability, performed using g:Profiler. The g:ProfileR web tool for functional profiling, g:GOST, was used to test the enrichment of the genes with significant heritability ($h^2_{\text{RV-gene}}$ p-value $< 2.75 \times 10^{-6}$), against gene-sets in common databases. The significant heritability genes for each phenotype were treated as separate gene lists for independent query, and statistical tests were conducted within a domain scope of only annotated genes (Methods). This table shows a list of statistically significant enriched terms for each gene list, corresponding to the phenotypes, adjusted for multiple testing using g:SCS (set counts and sizes) p-value < 0.05.
