# Supplementary material for: A method to estimate the contribution of rare coding variants to complex trait heritability
Source: Nat Commun. 2024 Feb 9;15:1245. doi: 10.1038/s41467-024-45407-8 (PMC10858280; doi:10.1038/s41467-024-45407-8)
Supplement: Supplementary file 9 — Reporting Summary [file 41467_2024_45407_MOESM9_ESM.pdf]

nature portfolio

Corresponding author(s): Dr. Guillaume Pare

Last updated by author(s): Dec 30, 2023

Reporting Summary

Nature Portfolio wishes to improve the reproducibility of the work that we publish. This form provides structure for consistency and transparency in reporting. For further information on Nature Portfolio policies, see our Editorial Policies and the Editorial Policy Checklist.

Statistics

For all statistical analyses, confirm that the following items are present in the figure legend, table legend, main text, or Methods section.

n/a	Confirmed	
☐	☒	The exact sample size (*n*) for each experimental group/condition, given as a discrete number and unit of measurement
☐	☒	A statement on whether measurements were taken from distinct samples or whether the same sample was measured repeatedly
☐	☒	The statistical test(s) used AND whether they are one- or two-sided<br>*Only common tests should be described solely by name; describe more complex techniques in the Methods section.*
☐	☒	A description of all covariates tested
☐	☒	A description of any assumptions or corrections, such as tests of normality and adjustment for multiple comparisons
☐	☒	A full description of the statistical parameters including central tendency (e.g. means) or other basic estimates (e.g. regression coefficient) AND variation (e.g. standard deviation) or associated estimates of uncertainty (e.g. confidence intervals)
☐	☒	For null hypothesis testing, the test statistic (e.g. *F*, *t*, *r*) with confidence intervals, effect sizes, degrees of freedom and *P* value noted<br>*Give P values as exact values whenever suitable.*
☒	☐	For Bayesian analysis, information on the choice of priors and Markov chain Monte Carlo settings
☒	☐	For hierarchical and complex designs, identification of the appropriate level for tests and full reporting of outcomes
☐	☒	Estimates of effect sizes (e.g. Cohen's *d*, Pearson's *r*), indicating how they were calculated

Our web collection on statistics for biologists contains articles on many of the points above.

Software and code

Policy information about availability of computer code

Data collection	Not applicable, data was was collected by UK Biobank (https://www.ukbiobank.ac.uk/)
Data analysis	Individual-level bio-bank genotype and phenotype data were downloaded from the UK Biobank and processed using PLINK version 1.9. Variant and gene-based annotations were conducted using the April 16, 2019 release of ANNOVAR. RARity can be run on all major platforms (e.g. GNU/Linux, macOS, Windows). For biobank-scale analyses, recommended hardware requirements are a unix-like virtual environment supporting a minimum of 250 GB RAM space for in-memory operations. Software requirements include the program dependencies: BASH (≥ 5.0) and R (≥ 3.6.0). Essential R dependencies include the packages: tidyverse and data.table. Detailed documentation, code, and step-by-step implementation of the methodology in the main text is available on the managed GitHub repository: (https://github.com/GMELab/RARity). The schematic diagram (Fig. 1) was generated with GraphPad Prism version 6.04 for Windows (https://www.graphpad.com).LD score regression (LDSC) and efficient mixed-model association method (BOLT-REML) were used to benchmark common variant heritability estimates. Further to estimating heritability with RARity, network and pathway analyses utilized g:Profiler, DisGenet and the Cytoscape 3.10.1 software.

For manuscripts utilizing custom algorithms or software that are central to the research but not yet described in published literature, software must be made available to editors and reviewers. We strongly encourage code deposition in a community repository (e.g. GitHub). See the Nature Portfolio guidelines for submitting code & software for further information.

Data

Policy information about availability of data

All manuscripts must include a data availability statement. This statement should provide the following information, where applicable:

- Accession codes, unique identifiers, or web links for publicly available datasets
- A description of any restrictions on data availability
- For clinical datasets or third party data, please ensure that the statement adheres to our policy

Individual genetic and phenotypic data were obtained from the UK Biobank (http://www.ukbiobank.ac.uk/), under application #15255. The UK Biobank study received approval from the National Health Service National Research Ethics Service North West. Access to the UK Biobank individual-level data is not publicly available and must be obtained via an application (https://www.ukbiobank.ac.uk/register-apply/).

UCSC genome browser (https://genome.ucsc.edu/) was utilized to access LiftOver for the conversion of Genome Reference Consortium Human Build 37 (GRCH37) to Genome Reference Consortium Human Build 38 built, and to obtain additional gene-level annotations such as gene-length, strand orientation and the gnomAD pLoF Metrics. Databases for gene-disease associations (DisGeNet, https://www.disgenet.org/), Drug Gene Interaction database (DGIdb, https://www.dgidb.org/) and HUGO Gene Nomenclature Committee (HGCN, https://www.genenames.org/) were utilized to inform on the importance of the target genes. Variant level annotations and pathogenicity scores, such as Mendelian Clinically Applicable Pathogenicity (M-CAP) Score, and Rare exome variant ensemble learner (REVEL) were obtained using ANNOVAR or downloaded directly from the web-based platform (Combined Annotation Dependent Depletion (CADD)).

This paper includes the source data for all main and supplemental figures.

Research involving human participants, their data, or biological material

Policy information about studies with human participants or human data. See also policy information about sex, gender (identity/presentation), and sexual orientation and race, ethnicity and racism.

Reporting on sex and gender	The terms sex (biological attribute) and gender (social and cultural circumstances) are used appropriately throughout the manuscript. 56% of the cohort were females and 44% were males as defined by genetic sex.
Reporting on race, ethnicity, or other socially relevant groupings	Unrelated, Caucasian individuals were chosen for the basis of this study as this was the largest single-ancestry cohort available in the UK Biobank.
Population characteristics	Cohort population characteristics are described in the "Study population" section of the methods : "The UK Biobank (UKB) study is a prospective cohort comprising of approximately 500,000 participants (ages 40–69 years) with extensive genotypic and phenotypic data from consenting individuals... Here we utilized two main genetic datasets from the UK Biobank. First, our primary source of RVs was the WES data with 17,975,236 variants on 200,643 participants (UKB data field: 23155). Second, CVs were extracted from imputed genotype data on 488,264 individuals (data field: 22418; v3 release).The acquisition and primary quality control (QC) of both genetic data are described elsewhere. Briefly, out of the 200,643 samples with WES data, individuals were excluded based on: consent withdrawal (n=11), call rates less than 99% (n=2), discordance between genetic and reported sex (n=18), a departure from putative ancestorial clusters based on the first two genetic principal components (n=3), assigned cluster membership to a continental population with less than 5,000 samples (n=12,765, of which, South Asian=3,395; African=3,168; Other=6,202), and 3rd degree or closer relatedness (n=14,156). In the remaining 173,688 individuals, an additional 6,340 were removed following QC of biomarker data (as described below). We focused on the 167,348 unrelated Caucasian participants to estimate narrow-sense heritability contributed by CVs, RVs, or the combined CV and RVs. "
Recruitment	UK Biobank recruitment was conducted through a longitudinal study design (https://www.ukbiobank.ac.uk/). No local recruitment of individuals was conducted.
Ethics oversight	UK Biobank has approval from the North West Multi-centre Research Ethics Committee (MREC) as a Research Tissue Bank (RTB) approval. Our research group has access to the UK Biobank under the application #15255.

Note that full information on the approval of the study protocol must also be provided in the manuscript.

Field-specific reporting

Please select the one below that is the best fit for your research. If you are not sure, read the appropriate sections before making your selection.

☒ Life sciences ☐ Behavioural & social sciences ☐ Ecological, evolutionary & environmental sciences

For a reference copy of the document with all sections, see nature.com/documents/nr-reporting-summary-flat.pdf

Life sciences study design

All studies must disclose on these points even when the disclosure is negative.

Sample size	Sample sizes were determined by the maximum size of a single-ancestry cohort available in the UKB with the following exclusion criteria as

Sample size	mentioned in the manuscript: "out of the 200,643 samples with WES data, individuals were excluded based on: consent withdrawal (n=11), call rates less than 99% (n=2), discordance between genetic and reported sex (n=18), a departure from putative ancestorial clusters based on the first two genetic principal components (n=3), assigned cluster membership to a continental population with less than 5,000 samples (n=12,765, of which, South Asian=3,395; African=3,168; Other=6,202), and 3rd degree or closer relatedness (n=14,156). In the remaining 173,688 individuals, an additional 6,340 were removed following QC of biomarker data (as described below). We focused on the 167,348 unrelated Caucasian participants to estimate narrow-sense heritability contributed by CVs, RVs, or the combined CV and RVs. "
Data exclusions	Genetic variants were called from WES data following the Functional Equivalent pipeline. All monomorphic variants (m=83,700), variants with missing genotypes in more than 10% samples (m=369,215), and those deviating significantly from Hardy-Weinberg Equilibrium (p-value < 5x10−6; m=35,317) were removed... Variants were annotated with MAF based on the UKB samples, as well as the 5 major ancestries identified in the Genome aggregation database (gnomAD 2.11): Latino, non-Finnish European, African/African American, East Asian, South Asian27. Qualifying RVs were defined as variants that were nonsynonymous single nucleotide variants, frameshift deletions or insertions, in-frame deletions or insertions, stop-gain, stop-loss and start-loss variants, with a minor allele count (MAC, the number of minor alleles at each locus in the population being studied) >2 and MAF below the cut-off (< 1%, <0.5% or <0.1%) in all gnomAD subpopulations, and locally in the UKB samples.
Replication	Genome-wide simulation analyses extensively tested for estimate reproducibility for 23 scenarios with 20 replicates or simulations per scenario. Each simulated outcome per scenario was generated by using the real UKB exome genotypes to capture real-world genetic architecture.
Randomization	Not applicable. Our study is an observational study design.
Blinding	Not applicable. Our study did not use any sort of intervention that would require blinding.

Reporting for specific materials, systems and methods

We require information from authors about some types of materials, experimental systems and methods used in many studies. Here, indicate whether each material, system or method listed is relevant to your study. If you are not sure if a list item applies to your research, read the appropriate section before selecting a response.

Materials & experimental systems

n/a	Involved in the study
☒	☐ Antibodies
☒	☐ Eukaryotic cell lines
☒	☐ Palaeontology and archaeology
☒	☐ Animals and other organisms
☒	☐ Clinical data
☒	☐ Dual use research of concern
☒	☐ Plants

Methods

n/a	Involved in the study
☒	☐ ChIP-seq
☒	☐ Flow cytometry
☒	☐ MRI-based neuroimaging

Plants

Seed stocks	*Report on the source of all seed stocks or other plant material used. If applicable, state the seed stock centre and catalogue number. If plant specimens were collected from the field, describe the collection location, date and sampling procedures.*
Novel plant genotypes	*Describe the methods by which all novel plant genotypes were produced. This includes those generated by transgenic approaches, gene editing, chemical/radiation-based mutagenesis and hybridization. For transgenic lines, describe the transformation method, the number of independent lines analyzed and the generation upon which experiments were performed. For gene-edited lines, describe the editor used, the endogenous sequence targeted for editing, the targeting guide RNA sequence (if applicable) and how the editor was applied.*
Authentication	*Describe any authentication procedures for each seed stock used or novel genotype generated. Describe any experiments used to assess the effect of a mutation and, where applicable, how potential secondary effects (e.g. second site T-DNA insertions, mosiacism, off-target gene editing) were examined.*
